# Construction of Single-Atom Catalysts for N, O Synergistic Coordination and Application to Electrocatalytic O_2_ Reduction

**DOI:** 10.3390/molecules28217264

**Published:** 2023-10-25

**Authors:** Jin-Hang Liu, Huixiong Jiang, Bokai Liao, Xiaohua Cao, Langhua Yu, Xiudong Chen

**Affiliations:** 1 Jiangxi Province Engineering Research Center of Ecological Chemical Industry, School of Chemistry and Chemical Engineering, Jiujiang University, Jiujiang 332005, China; ljh2016hust@126.com (J.-H.L.); 6080133@jju.edu.cn (H.J.); caojimmy@126.com (X.C.); 13767810834@163.com (L.Y.); 2 Guangzhou Key Laboratory for Clean Energy and Materials, School of Chemistry and Chemical Engineering, Guangzhou University, Guangzhou 510006, China; bokailiao@gzhu.edu.cn

**Keywords:** single-atom catalyst, electrocatalysis, oxygen reduction reaction, density functional theory, limiting potential

## Abstract

Replacing expensive platinum oxygen reduction reaction (ORR) catalysts with atomically dispersed single-atom catalysts is an effective way to improve the energy conversion efficiency of fuel cells. Herein, a series of single-atom catalysts, TM-N_2_O_2_C_x_ (TM=Sc-Zn) with TM-N_2_O_2_ active units, were designed, and their catalytic performance for electrocatalytic O_2_ reduction was investigated based on density functional theory. The results show that TM-N_2_O_2_C_x_ exhibits excellent catalytic activity and stability in acidic media. The eight catalysts (TM=Sc, Ti, V, Cr, Mn, Fe, Co, and Ni) are all 4e^−^ reaction paths, among which Sc-N_2_O_2_C_x_, Ti-N_2_O_2_C_x_, and V-N_2_O_2_C_x_ follow dissociative mechanisms and the rest are consistent with associative mechanisms. In particular, Co-N_2_O_2_C_x_ and Ni-N_2_O_2_C_x_ enable a smooth reduction in O_2_ at small overpotentials (0.44 V and 0.49 V, respectively). Furthermore, a linear relationship between the adsorption free energies of the ORR oxygen-containing intermediates was evident, leading to the development of a volcano plot for the purpose of screening exceptional catalysts for ORR. This research will offer a novel strategy for the design and fabrication of exceptionally efficient non-precious metal catalysts on an atomic scale.

## 1. Introduction

Fuel cells (FCs) have garnered much attention as a technology for converting chemical energy into electrical energy because of their high energy conversion efficiency, high energy density, and non-polluting characteristics [1,2]. Compared with the anode reaction, the oxygen reduction reaction (ORR) at the cathode is kinetically slow, and in-depth research on the ORR process is of great significance for improving the overall performance of FCs [3,4,5]. Superior catalysts can be effective in solving the bottleneck problem of ORR. Among the many developed catalysts, Pt and its alloys are considered the best ORR catalysts for their high current density and low initial voltage. However, the scarcity of Pt resources, expensive price, poor durability, and easy deactivation limit its large-scale commercial application. Therefore, developing ORR electrocatalysts with a high catalytic activity, cheap availability, and low overpotential has recently been a hot research topic [6,7,8].

Single-atom catalysts (SACs) can achieve a high dispersion of metal atoms, which not only maximizes the utilization of metal atoms but also significantly improves the catalytic activity of the catalyst [9,10,11,12]. Since Pt_1_/FeO_x_ single-atom catalysts were first reported by Zhang et al., a series of SACs have been notified and widely used electrocatalysis [13]. Guo et al. investigated the catalytic performance of a transition metal-anchored N-doped graphene single-atom catalyst (TMN_x_-GR) for electrocatalytic CO_2_ reduction based on density functional theory [14]. The results showed that TMN_x_-GR exhibited good catalytic activity, with NiN_4_-GR showing the best selectivity for the product CO. Chen’s group successfully constructed single-atom Cu-containing catalysts on N-doped carbon nanosheets and applied them to overall water splitting [15]. The results indicate that the oxygen evolution reaction at 200 mV and the hydrogen evolution reaction at 216 mV was achieved at a current density of 10 mA·cm^−2^, providing a new strategy for constructing non-precious metal catalysts. Wang et al. investigated the potential of transition metal-embedded g-C_4_N_3_ (TM@g-C_4_N_3_) single-atom catalysts for nitrogen reduction reactions (NRR) by a high-throughput screening system. Among the 30 candidate materials [4], V@g-C_4_N_3_ was identified as the most active NRR catalyst, with a limiting potential of −0.37 V. This work leads the way for the construction of efficient NRR single-atom catalysts using g-C_4_N_3_ as a novel carrier. Yi and co-workers prepared atomically dispersed Fe-N_x_ species (Fe loading up to 8.3 wt %) on porous porphyrin triazine-based frameworks (FeSAs/PTF) using a simple isothermal method [16]. FeSAs/PTF-600 has a high density of single-atom Fe-N_4_ active sites and a high layered porosity, and good electrical conductivity, resulting efficient activity, methanol resistance, and ORR superstability under alkaline and acidic conditions. Hunter et al. theoretically investigated the catalytic performance of N-doped graphene single-atom catalysts (M_1_M_2_@N_6_V_4_ and M_1_M_2_@N_8_V_4_, M = Co, Pt, Fe, Ni) for ORRs [17]. It was found that CoPt@N_8_V_4_ exhibited the most desirable ORR catalytic activity (*ƞ* = 0.30 V), and a basis for the screening of highly active ORR catalysts was proposed by volcano mapping.

Most of the reported ORR SACs are of metal–N_4_ coordination [18,19]. However, recent studies have found that single-atom catalysts with N and O co-coordination with metal atoms exhibit excellent catalytic activity, even better than metal–N_4_ coordination catalysts. Ge et al. investigated the catalytic performance of the oxygen reduction reaction (ORR) of M-N_4-x_O_x_ (M=Fe, Co, and Ni; x = 1–4) in detail based on density functional theory [20]. The results show that Co-N_3_O_1_ and Ni-N_2_O_2_ exhibit the best catalytic activity, with overpotentials of 0.27 and 0.32 V, respectively, which is significantly better than the Pt catalysts. Electronic structure and density of states analyses revealed that one of the reasons for the higher activity of Co-N_3_O_1_ and Ni-N_2_O_2_ is their small energy gap. O doping can improve the electronic structure of the original catalyst, which can adjust the adsorption capacity of ORR intermediates. Dong et al. successfully synthesized a low-Mn-content single-atom catalyst (Mn-NO/CNs) with Mn-N_2_O_2_ sites, which exhibited good activity in the catalysis of CO_2_ [21]. The results show that the addition of oxygen atoms changed the coordination environment surrounding the Mn atoms, modifying the electronic structure of the catalyst and improving the catalytic efficiency of Mn-NO/CNs for CO_2_ reduction. Wei et al. designed seven two-dimensional (2D) metal–organic framework (MOF) materials with TMN_2_O_2_ ligand units and conducted theoretical investigations into their catalytic activities towards oxygen reduction and evolution reaction (ORR and OER). The findings demonstrate that CoN_2_O_2_ manifests superior catalytic performance, displaying low overpotential values of 0.33 V and 0.30 V for ORRs and OERs, respectively [22]. The above reports open up new avenues for the development of ORR catalysts.

Although TM-N_2_O_2_ active units have been reported as catalytic materials for ORR applications, it has not been explicitly addressed whether the catalytic activity of TM-N_2_O_2_ active units would be significantly influenced by ligand variations. Based on this, we devised a series of TM-N_2_O_2_ (TM=Sc, Ti, V, Cr, Mn, Fe, Co, Ni, Cu, Zn) coordination-type single-atom catalysts (TM-N_2_O_2_C_x_) by employing 4-hydroxy benzonitrile as the ligand and investigated their catalytic activities and reaction mechanisms for electrocatalytic O_2_ reduction in an acidic environment. The comprehensive analysis and discussion of the stability, reaction pathway, overpotentials, and electronic structure of TM-N_2_O_2_C_x_ elucidate that a majority of the catalysts exhibit remarkable catalytic activity. Furthermore, a comparative analysis of the work conducted by Wei et al [22]. revealed that changing the ligand does indeed exert an effect on the catalytic activity of the TM-N_2_O_2_ site, but without altering the trend of the volcano curve. These findings will serve as a stimulus for further experimental and theoretical explorations in ORRs.

## 2. Results and Discussion

### 2.1. The Structural Features of TM-N_2_O_2_C_x_ Single-Atom Catalysts

**Figure 1:**
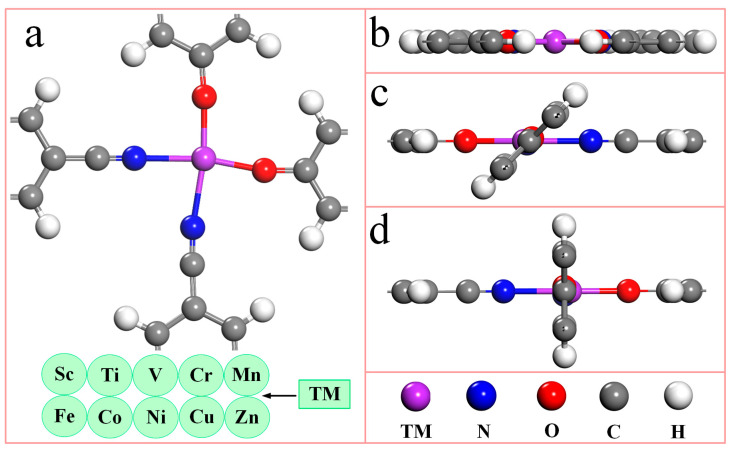
The optimized geometric structures of TM-N_2_O_2_C_x_ single-atom catalysts. (**a**) Top view of unit cell. (**b**–**d**) Side views of unit cell. The purple, blue, red, gray, and white spheres represent TM, N, O, C, and H atoms, respectively. TM represents 10 metal atoms of the first transition metal series.

Figure 1 is the top (1a) and side views (1b-d) of the unit cell structure model of the TM-N_2_O_2_C_x_ single-atom catalyst. It can be seen from Figure 1a that the unit cell of the TM-N_2_O_2_C_x_ single-atom catalyst contains one transition metal atom, two nitrogen atoms, two oxygen atoms, fourteen carbon atoms, and eight hydrogen atoms, and each transition metal atom coordinates with two nitrogen atoms and two oxygen atoms simultaneously. Due to the different orientations of ligands in the bonding process, the TM-N_2_O_2_C_x_ single-atom catalyst has three initial configurations, as shown in Figure 1b–d, respectively. The structural optimization of ten SACs of the first transition metal series (Table S1) offering the stable configurations of Sc-N_2_O_2_C_x_ and Cu-N_2_O_2_C_x_ are demonstrated in Figure 1b,d, respectively. Structural optimization of ten single-atom catalysts (SACs) from the first transition metal series (refer to Table S1) reveals that stable configurations of Sc-N_2_O_2_C_x_ and Cu-N_2_O_2_C_x_ are illustrated in Figure 1b and 1d, respectively. In comparison, the regular structures of the remaining eight catalysts are shown in Figure 1c.

A Hirshfeld charges analysis shows that the metal atoms in the ten SACs all have partial positive charges [23], while the N and O atoms coordinated with them all have partial negative charges (Table S1), which indicates that there is charge transfer between the metal atoms and their neighboring atoms in the process of binding with the ligand so that the metal atoms can effectively bind with the ligand. Figure S1 is the projected partial density of states of the TM-N_2_O_2_C_x_ SACs. The green line is the 3d orbit of the metal atoms, while the blue and red lines are the 2p orbitals of the N and O atoms, respectively. The orbit’s degree of overlap can reflect the interatomic interaction’s strength. The better the overlap, the stronger the interaction; on the contrary, the weaker it is. Figure S1 shows that the 3d orbitals of the metal atoms and the N and O atoms in the ten poor catalysts overlap very well, proving that metal atoms have a strong coordination ability with N and O atoms in ligands. In addition, except for the metal atoms in Sc-N_2_O_2_C_x_, Ni-N_2_O_2_C_x_, and Zn-N_2_O_2_C_x_, the metal atoms in the other SACs have magnetism and the Mn atom possesses the highest magnetic moment of 4.504 μB, which may also affect the catalytic activity of the catalysts.

### 2.2. The Structural Stability of TM-N_2_O_2_C_x_

**Figure 2:**
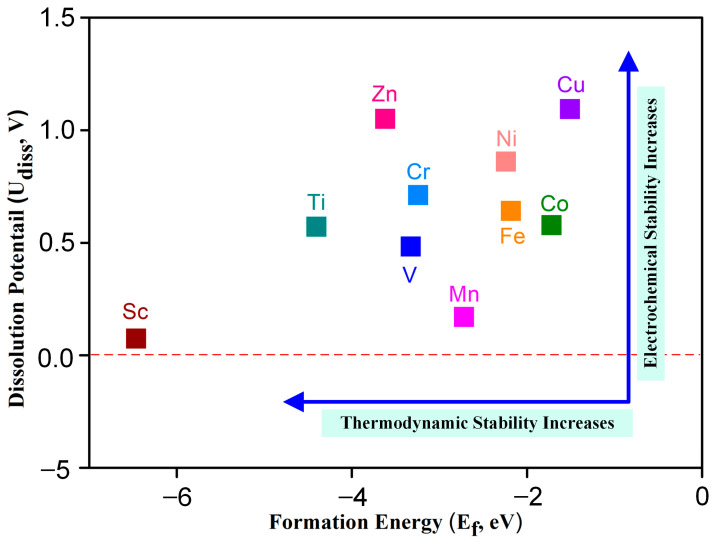
Dissolution potential and formation energy of transition metal atoms in TM-N_2_O_2_C_x_, the dash line represents a potential of 0 V.

As the structural stability of the catalyst plays an essential role in maintaining the catalytic activity of the motivation, the thermodynamic and electrochemical stability of TM-N_2_O_2_C_x_ were studied according to the formation energy (E_f_) and dissolution potential (U_diss_) [24], respectively. E_f_ and U_diss_ can be obtained from the formulas E_f_ = E_TM-N_2_O_2_C_x__ − E_TM_ − E_N_2_O_2_C_x__ (1) and U_diss_ = U°_diss_ − E_f_/ne (2) (detailed data are shown in Table S2), where E_TM-N_2_O_2_C_x__, E_TM_, and E_N_2_O_2_C_x__ are the total energies of TM-N_2_O_2_C_x_, TM, and N_2_O_2_C_x_, respectively. U°_diss_ and n are the standard dissolution potential and the number of electrons involved in the dissolution of transition metals, respectively. The negative value of formation energy indicates that it is an exothermic reaction in the process of a metal atom combining with a ligand to form a single-atom catalyst; so, the more negative the value of formation energy is, the stronger the thermodynamic stability of the catalyst is and vice versa. Figure 2 shows that the formation energies of ten kinds of SACs are negative, indicating that TM-N_2_O_2_C_x_ SACs offer good thermodynamic stability.

During the process of electrocatalysis, if the dissolution potential of metal atoms in the single-atom catalyst is relatively low, it becomes easier for these metal atoms to undergo oxidation and subsequently dissolve into the solvent. This phenomenon ultimately leads to a substantial decrease in the overall catalytic activity exhibited by the catalyst. Consequently, in order to enhance the stability of the catalyst, it is essential for the metal atoms within it to possess a higher dissolution potential. The dissolution potentials of metal atoms for all ten catalysts investigated in Figure 2 were found to be greater than 0 V. Notably, these values surpass the standard dissolution potentials of the respective metals (refer to Table S2), thereby indicating the exceptional electrochemical stability displayed by the ten TM-N_2_O_2_C_x_ single-atom catalysts that were examined in this study.

### 2.3. O_2_ Adsorption

**Figure 3:**
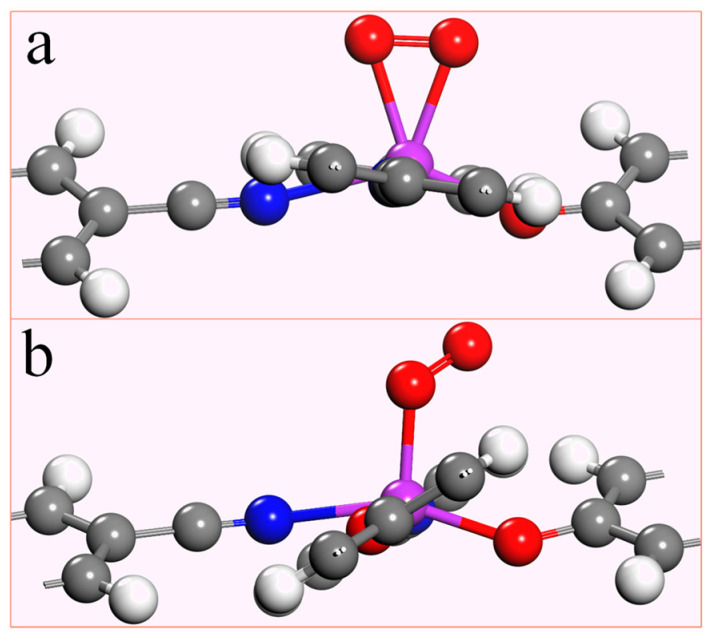
Two different adsorption states of O_2_ on TM-N_2_O_2_C_x_,(**a**) side-on configuration, (**b**) end-on configuration.

Since the electrocatalytic oxygen reduction reaction occurs in an aqueous solution, the O_2_ molecule must be effectively adsorbed by the catalyst for the response to happen smoothly. To investigate the adsorption capacity of the TM-N_2_O_2_C_x_ single-atom catalyst for O_2_, the adsorption energy (taking O_2_ adsorption as an example) was obtained by the formula: E_ads_ = E_TM-N_2_O_2_C_x_-O_2__ − E_TM-N_2_O_2_C_x__ − E_O_2__ (3). E_TM-N_2_O_2_C_x_-O_2__ is the total energy of the adsorbed O_2_ and TM-N_2_O_2_C_x_, while E_TM-N_2_O_2_C_x__ and E_O_2__ are the total energy of TM-N_2_O_2_C_x_ and the single O_2_ molecule. If the adsorption energy is negative, indicating that O_2_ can be effectively adsorbed, and the more negative the value, the stronger the adsorption is. There are two forms of O_2_ adsorption on the catalyst, namely end-on configuration and side-on configuration (Figure 3). The most stable adsorption states and adsorption energies of O_2_ on ten single-atom catalysts of the first transition metal series are listed in Table S3, which shows that the stable adsorption states of O_2_ on Sc-N_2_O_2_C_x_, Ti-N_2_O_2_C_x_, and V-N_2_O_2_C_x_ are side-on configuration (Figure 3a). At the same time, the remaining seven are end-on configuration (Figure 3b). Additionally, the adsorption energies of the ten stable states are in the range of −4.22 ~ −0.39 eV, which indicates that O_2_ can be effectively adsorbed on the catalyst to facilitate the reduction reaction.

### 2.4. ORR Catalytic Performance

#### 2.4.1. Selectivity of Reaction (2e^−^ or 4e^−^)

**Figure 4:**
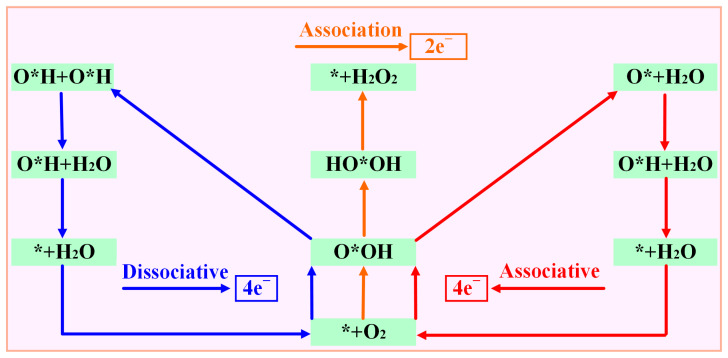
Possible reaction path of oxygen reduction reaction.

According to the different reduction products, the ORR can be divided into a 2e^−^ pathway to produce H_2_O_2_ and a 4e^−^ pathway in which the product is H_2_O. The 4e^−^ pathway can be divided into a dissociative mechanism and association mechanism (Figure 4). Compared with the 2e^−^ pathway, the 4e^−-^ pathway has better energy conversion efficiency, so suitable ORR catalysts must have good 4e^−^ selectivity. The intermediate O*OH (* + O_2_ + H^+^ + e^−^ → O*OH) is obtained by one-step protonation of O_2_. O*OH can be reduced to H_2_O_2_ through the path O*OH + H^+^ + e^−^ → * + H_2_O_2_ or O*OH + H^+^ + e^−^ →O* + H_2_O to obtain O*; so, it is necessary to determine whether the catalyst is thermodynamically inclined to the 4e^−^ pathway or the 2e^−^ pathway. Therefore, this paper is determined by comparing the formation barrier of H_2_O_2_ and the barrier to obtain O* and H_2_O.

**Figure 5:**
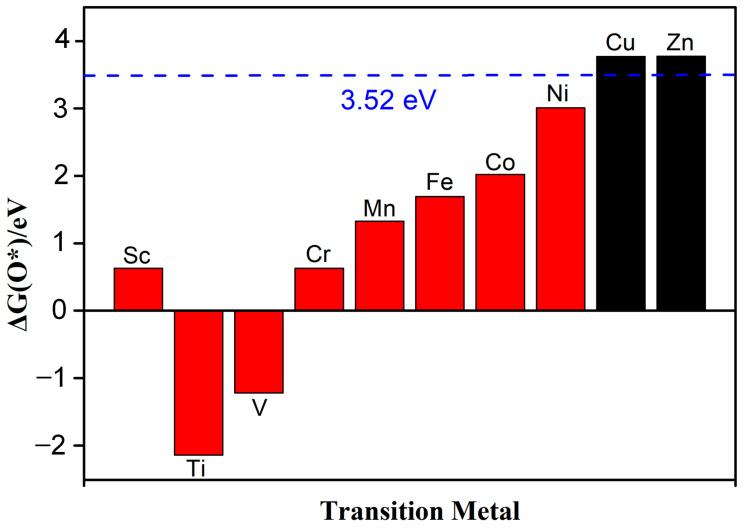
The Gibbs free energy of ∆G(O*), and the dotted line represents the free energy value of 3.52 eV, which is obtained by G (O *) = G (H_2_O_2_) − G (H_2_O).

Guo et al. systematically studied the selectivity of ORRs and found that the Gibbs free energy (∆G_O*_) of O* for the 4e^−^ pathway should be less than 3.52 eV (∆G_H_2_O_2__ − ∆G_H_2_O_) [25]. On the contrary, the Gibbs free energy for the 2e^−^ pathway should be greater than 3.52 eV. The Gibbs free energy of the reaction was determined using the computational hydrogen electrode model (CHE) proposed by Nørskov and co-workers [26], and the details are described in our previous reports [27]. This paper focuses on reactions under strongly acidic conditions (pH = 0). Figure 5 shows that except for Cu-N_2_O_2_C_x_ and Zn-N_2_O_2_C_x_, the Gibbs free energy of ∆G_*O_ for the other eight catalysts is all less than 3.52 eV, indicating that the ORR mechanism of Cu-N_2_O_2_C_x_ and Zn-N_2_O_2_C_x_ is the 2e^−^ pathway (Figure S2), and the rest of the SACs are more inclined to the 4e^−^ pathway. Next, we focus on exploring the 4e^−^ pathway mechanism of the above eight catalysts.

#### 2.4.2. 4e^−^ Pathway

**Figure 6:**
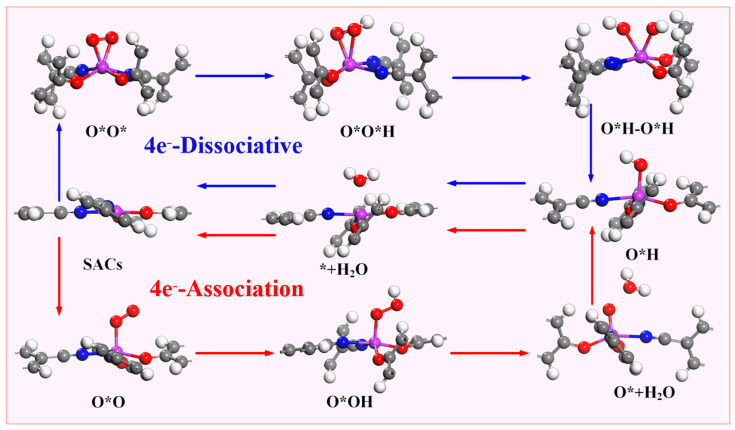
Two 4e^−^ pathways and corresponding intermediates of oxygen reduction reaction.

As previously mentioned, the 4e^−^ pathway encompasses both associative and dissociative mechanisms, with the specific reaction mechanism employed in the 4e^−^ process being dependent on the stable adsorption configuration of O_2_ on the catalyst. Figure 6 demonstrates that when the stable adsorption configuration of O_2_ is side-on, the 4e^−^ reaction tends to proceed via the dissociative mechanism, whereas when the stable adsorption configuration of O_2_ is end-on, the 4e^−^ reaction follows the associative mechanism. Due to the fact that the stabilization of the adsorption configuration on Sc-N_2_O_2_C_x_, Ti-N_2_O_2_C_x_, and V-N_2_O_2_C_x_ is side-on, the electrocatalytic O_2_ reduction on these catalysts adopts the 4e^−^ dissociative mechanism. In contrast, the five catalysts from Cr- N_2_O_2_C_x_ to Ni- N_2_O_2_C_x_ exhibit associative mechanisms for the 4e^−^ pathway.

**Figure 7:**
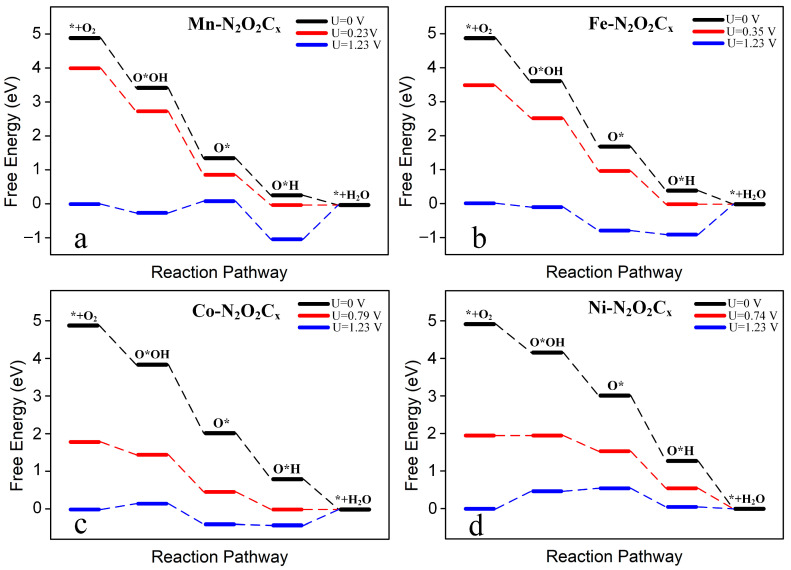
Free energy diagram of 4e^−^ ORR on Mn-N_2_O_2_C_x_ (**a**), Fe-N_2_O_2_C_x_ (**b**), Co-N_2_O_2_C_x_ (**c**), and Ni-N_2_O_2_C_x_ (**d**) under different potentials. The black line, blue line, and red line represent U = 0 V, U = limiting potential, and U = 1.23 V, respectively.

Figure 7 and Figure S3 show the free energy diagrams of the 4e^−^ pathway of electrocatalytic O_2_ reduction for the eight TM-N_2_O_2_C_x_ single-atom catalysts at three different applied potentials. The black and blue lines represent the applied potentials of 0 V and 1.23 V, respectively, while the red line represents the limiting potential. Figure 7 shows that each step of the protonation process of O_2_ reduction by Mn-N_2_O_2_C_x_, Fe-N_2_O_2_C_x_, Co-N_2_O_2_C_x_, and Ni-N_2_O_2_C_x_ without applied potential is exothermic; however, the final step of protonation (O*H + H^+^ + e^−^ → * + H_2_O) in the 4e^−^ reaction catalyzed by Sc-N_2_O_2_C_x_, Ti-N_2_O_2_C_x_, V-N_2_O_2_C_x_, and Cr-N_2_O_2_C_x_ is an endothermic reaction (Figure S3).

The rate-determining step was determined by comparing the maximum increase in the protonation-free energy in the 4e^−^ reaction. It was found that except for Ni-N_2_O_2_C_x_, of which the rate-determining step was * + O_2_ + H^+^ + e^−^ → O*OH (Figure 7d), the remaining seven catalysts were all O*H + H^+^ + e^−^ → * + H_2_O (Table 1). The limiting potential (U_L_) of the ORR can be determined according to the formula U_L_ = −ΔG_max_/ne (4), where ΔG_max_ and n are the increase in the free energy in the rate-determining step and the number of electrons transferred in the reaction, respectively. Finally, the overpotential (*ƞ*) of the ORR was determined by the difference between U_L_ and 1.23 V (*ƞ =* U_L_ − 1.23 V), and the detailed data statistics are listed in Table 1.

Among the eight SACs investigated, the limiting potentials of Mn-N_2_O_2_C_x_, Fe-N_2_O_2_C_x_, Co-N_2_O_2_C_x_, and Ni-N_2_O_2_C_x_ are all greater than 0 V. At the same time, Sc-N_2_O_2_C_x_, Ti-N_2_O_2_C_x_, V-N_2_O_2_C_x_, and Cr-N_2_O_2_C_x_ display limiting potentials lower than 0 V; especially, Sc-N_2_O_2_C_x_, Ti-N_2_O_2_C_x_, and V-N_2_O_2_C_x_ are all lower than −0.80 V, which results in their overpotentials all being greater than 2 V, meaning that more external potentials need to be applied for the occurrence of the ORR. In comparison, the limiting potential values of the other five catalysts are relatively positive, lowering the corresponding overpotential. With the exception of Cr-N_2_O_2_C_x_, whose overpotential exceeds 1 V, the overpotentials of the other catalysts are all lower than 1 V. Notably, Co-N_2_O_2_C_x_ and Ni-N_2_O_2_C_x_ possess limiting potentials of 0.79 V and 0.74 V, respectively, leading to overpotentials below 0.50 V, which means that the ORR catalytic performance of Co-N_2_O_2_C_x_ and Ni-N_2_O_2_C_x_ is comparable to that of Pt catalysts with a working potential of 0.78 V [26] and is better than that of FeN_4_-doped graphene (0.35 V) [28], The above results show that Co-N_2_O_2_C_x_ and Ni-N_2_O_2_C_x_ are expected to be promising ORR catalysts.

### 2.5. Scaling Relationship between Oxygen-Containing Intermediates

The catalytic performance of the catalyst is determined by its electronic structure. According to Sabatier’s principle, an excellent catalyst has appropriate adsorption capacity for reaction intermediates, which is neither strong nor weak. If the catalyst’s adsorption capacity is too high, it prevents intermediate detachment, causing the catalytic active site to become passivated and, finally, leads to a considerable loss in catalytic performance [29]. Conversely, if the adsorption capacity is too weak, the adsorption of intermediates on the catalyst surface is not favorable, making the reaction unable to proceed. Therefore, if there is a relationship between the adsorption energies of the ORR intermediates, we can effectively discover and design the most suitable ORR catalysts by the descriptor-based method.

**Figure 8:**
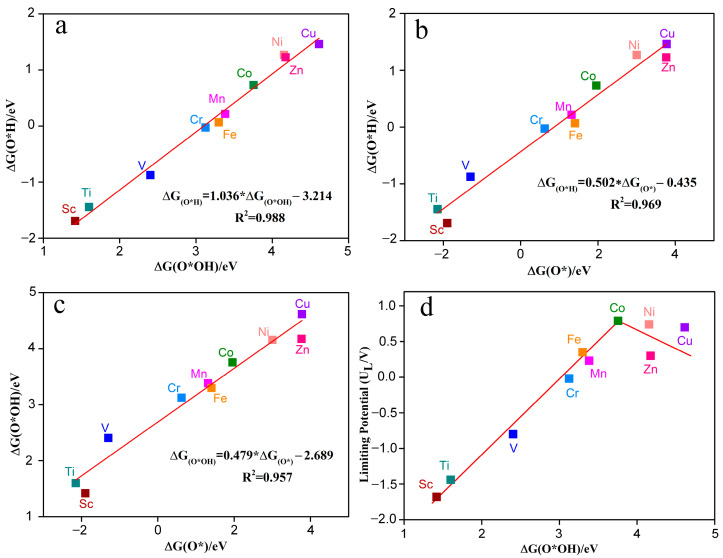
The Gibbs free energy of intermediates O*OH and O*H (**a**), O* and O*H (**b**), O* and O*OH (**c**) on the catalyst of TM-N_2_O_2_C_x_. (**d**) Volcano plots between limiting potential and ∆G(O*OH).

Figure 8a–c shows the Gibbs adsorption free energy relationships between different reaction intermediates (such as O*OH, O*H, O*) for the electrocatalytic O_2_ reduction by TM-N_2_O_2_C_x_ single-atom catalysts. The Gibbs adsorption free energy relative to H_2_O and H_2_ were calculated based on the following equations: ΔG_O*H =_ G_O*H_ + 0.5G_H2_ − G* − G_H2O_ (6); ΔG_O* =_ G_O*_ + G_H2_ − G* − G_H2O_ (7); ΔG_O*OH =_ G_O*OH_ + 1.5G_H2_ − G* − 2G_H2O_ (8). G* is the free energy of TM-N_2_O_2_C_x_, while G_H2_ and G_H2O_ represent the gas phase free energy of H_2_ and H_2_O molecules, respectively. The results indicate that no matter the active metal atom, there is a universal scaling relation between O*OH, O*H, and O*. ΔG_O*H_ can be represented by ΔG_O*OH_ through the function ΔG_O*H_ = 1.036ΔG_O*OH_ − 3.214 eV (Figure 8a) or by ΔG_O*_ through the function ΔG_O*H_ = 0.502ΔG_O*_ − 0.435 eV (Figure 8b). The coefficients of determination (R^2^) are 0.988 and 0.969, respectively, indicating a very strong linear relationship between ΔG_O*H_ and ΔG_O*OH_ or ΔG_O*_. In addition, Δ G_O*OH_ can be created by Δ G_O*_ through the function Δ G_O*OH_ = 0.479 Δ G_O*_ −2.689 eV (Figure 8c), and the same strong linear relationship exists between ΔG_O*OH_ and ΔG_O*_ (R^2^ = 0.957). These results are in agreement with previous studies [30,31]. Thus, the catalytic activity of TM-N_2_O_2_C_x_ single-atom catalysts for electrocatalytic O_2_ reduction can be described by the descriptors ΔG_O*H_, ΔG_O*OH_, or ΔG_O*_.

Figure 8d shows that the adsorption strength of TM-N_2_O_2_C_x_ to O*OH (ΔG_O*OH_) significantly affects the limiting potential of ORR. With the increasing value of ΔG_O*OH_, the limiting potential (U_L_) shows a trend of increasing and then decreasing, and there is a significant volcanic relationship between ΔG_O*OH_ and U_L_. Among the ten TM-N_2_O_2_C_x_ single-atom catalysts studied in this paper, the limiting potentials of Co-N_2_O_2_C_x_ and Ni-N_2_O_2_C_x_ are very close to the top of the volcano diagram, making their limiting potential values closest to the potential equilibrium value of the ORR (1.23 V) and allowing a small overpotential of 0.44 V and 0.49 V, respectively (Table 1), which is consistent with the overpotential of commercial Pt (0.45 V). In contrast, the limiting potentials of Sc-N_2_O_2_C_x_, Ti-N_2_O_2_C_x_, and V-N_2_O_2_C_x_ are far from the top of the volcano plot, leading to an ORR with higher overpotentials (>2 V). Therefore, the volcano curve can effectively help us to screen for superior ORR catalysts. 

## 3. Computational Methods

This study is carried out based on the density functional theory of spin polarization with the help of the Dmol^3^ module [32]. The structure optimization, total energy, and electronic properties of the TM-N_2_O_2_C_x_ models are treated using the Perdew–Burke–Ernzerhof (PBE) exchange-related energy generalization in the generalized gradient approximation (GGA) [33], the basis group is used as a double numerical plus polarization basis group, and the core electrons are treated in an all-electron way. To obtain a high accuracy, the energy convergence criterion is 10^−6^ Ha, the Monkhorst–Pack grid uses 5 × 5 × 1 K points for structure optimization and 10 × 10 × 1 for performance calculation, and the vacuum layer is set to 15 Å to eliminate the interaction between TM-N_2_O_2_C_x_ layers. In addition, Van der Waals dispersion was introduced to better describe the adsorption of O_2_ and reaction intermediates on the TM-N_2_O_2_C_x_ surface. Since the ORR occurs in solution, a conductor approximation shielding model (COSMO) is usually used for electrocatalytic reactions to better simulate the real reaction environment, and the dielectric constant of the solvent is set to 78.54 in this paper [34].

## 4. Conclusions

We designed a single-atom catalyst (TM-N_2_O_2_C_x_) with a TM-N_2_O_2_ coordination unit and investigated its catalytic performance for electrocatalytic O_2_ reduction based on density functional theory. The obtained results demonstrate the outstanding thermodynamic and electrochemical stability of TM-N_2_O_2_C_x_. Notably, most catalysts examined in this study follow the 4e^−^ pathway for O_2_ reduction, with the exception of Cu-N_2_O_2_C_x_ and Zn-N_2_O_2_C_x_ which exhibit a 2e^−^ ORR pathway. Sc-N_2_O_2_C_x_, Ti-N_2_O_2_C_x_, and V-N_2_O_2_C_x_ conform to the dissociative mechanism, while the rest follow the associative mechanism. Among the ten catalysts studied in this paper, Co-N_2_O_2_C_x_ and Ni-N_2_O_2_C_x_ display the lowest overpotential (less than 0.5 V), which is equivalent to the overpotential of the benchmark catalyst Pt, making Co-N_2_O_2_C_x_ and Ni-N_2_O_2_C_x_ possible as alternative candidates to Pt. It is found that there is a scaling relationship between the adsorption-free energy of oxygen-containing intermediates (O*OH, O*H, O*) and a volcano curve between the adsorption-free energy of O*OH and the limiting potential, providing a foundation for the selection of exceptional ORR catalysts.

## Figures and Tables

**Table 1:** The calculated rate-determining steps, limiting potentials (U_L_/V), and over-potential (***ƞ***/V) for the ORR of the TM-N_2_O_2_C_x_ SACs are listed.

TM-N_2_O_2_C_x_	PDS	U_L_	*ƞ*
**Sc-N_2_O_2_C_x_**	*OH → * + H_2_O	−1.68	2.91
**Ti-N_2_O_2_C_x_**	*OH → * + H_2_O	−1.44	2.67
**V-N_2_O_2_C_x_**	*OH → * + H_2_O	−0.80	2.03
**Cr-N_2_O_2_C_x_**	*OH → * + H_2_O	−0.02	1.25
**Mn-N_2_O_2_C_x_**	*OH → * + H_2_O	0.23	1.00
**Fe-N_2_O_2_C_x_**	*OH → * + H_2_O	0.35	0.88
**Co-N_2_O_2_C_x_**	*OH → * + H_2_O	0.79	0.44
**Ni-N_2_O_2_C_x_**	* + O_2_ → O*OH	0.74	0.49
**Cu-N_2_O_2_C_x_**	* + O_2_ → O*OH	0.70	0.53
**Zn-N_2_O_2_C_x_**	O*OH→ *+H_2_O_2_	0.30	0.93

## Data Availability

Data are contained within the article.

## Supplementary Materials

The following supporting information can be downloaded at: https://www.mdpi.com/article/10.3390/molecules28217264/s1. Figure S1. Projected partial density of states (PDOS) of TM-N_2_O_2_C_x_, (a) Sc-N_2_O_2_C_x_, (b) Ti-N_2_O_2_C_x_, (c) V-N_2_O_2_C_x_, (d) Cr-N_2_O_2_C_x_, (e) Mn-N_2_O_2_C_x_, (f) Fe-N_2_O_2_C_x_, (bg) Co-N_2_O_2_C_x_, (bh) Ni-N_2_O_2_C_x_,(i)Cu-N_2_O_2_C_x_, (j)Zn-N_2_O_2_C_x_. Figure S2. Free energy diagram of 2e^−^ ORR on Cu-N_2_O_2_C_x_ (a) and Zn-N_2_O_2_C_x_ (b) under 0 V. Figure S3. Free energy diagram of 4e^−^ ORR on Sc-N_2_O_2_C_x_ (a), Ti-N_2_O_2_C_x_ (b), V-N_2_O_2_C_x_ (c), and Cr-N_2_O_2_C_x_ (d) under different potentials. Table S1 The lattice constants, top, and side views of optimized crystal structures of TM-N_2_O_2_C_x_. Table S2 Computed formation energy (E_f_) and dissolution potential (U_diss_) of TM-N_2_O_2_C_x_. Table S3. The top and side views of the most stable adsorption state of O_2_ on TM-N_2_O_2_C_x_. Table S4. The calculated free energy corrections (Gc) for adsorbates.
