# Supplementary material for: Construction of Single-Atom Catalysts for N, O Synergistic Coordination and Application to Electrocatalytic O2 Reduction
Source: Molecules. 2023 Oct 25;28(21):7264. doi: 10.3390/molecules28217264 (PMC10650445; doi:10.3390/molecules28217264)
Supplement: Supplementary file 1 [file molecules-28-07264-s001.zip › molecules-2672337-supplementary.pdf]

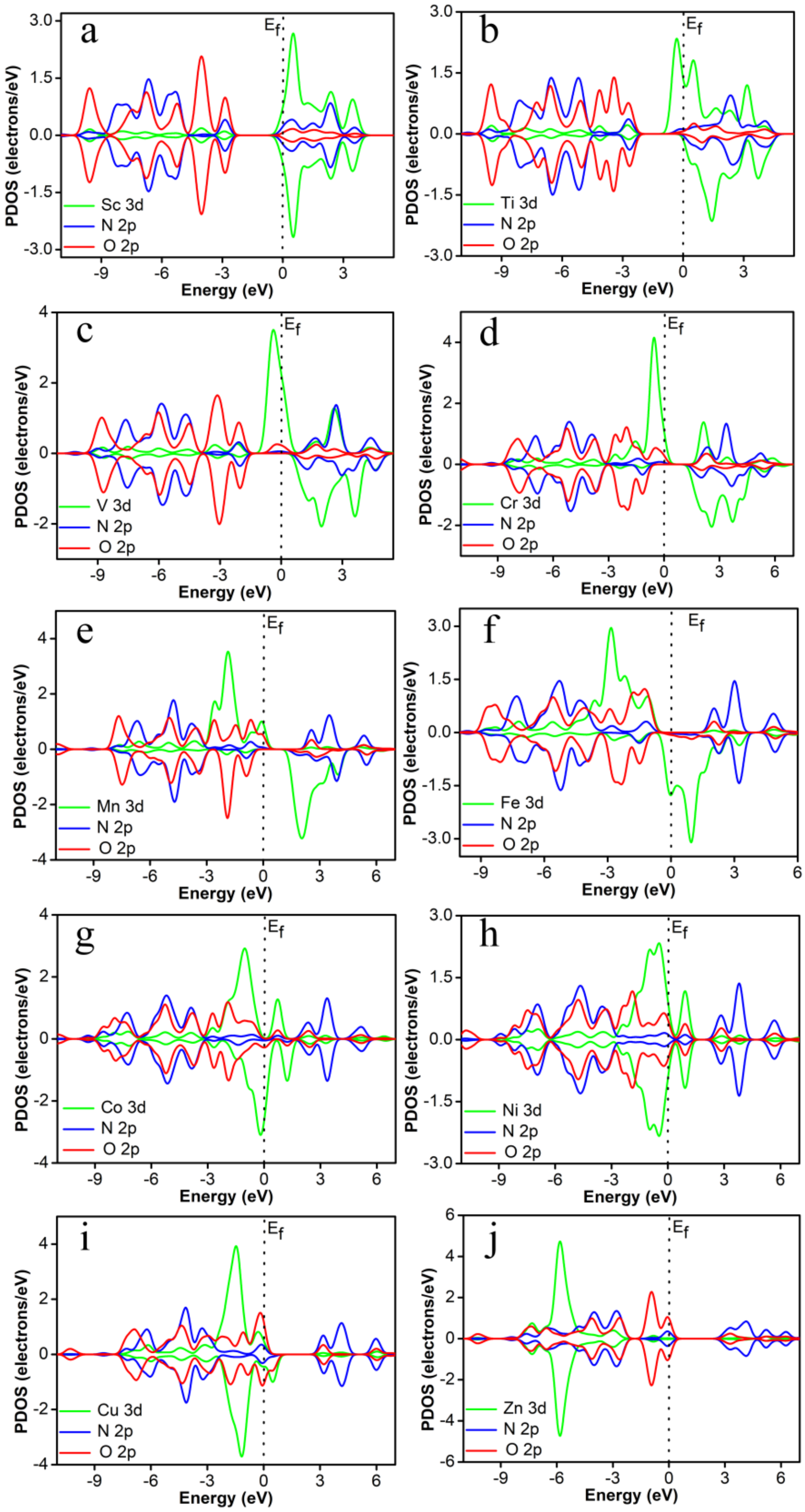

Figure S1. Projected partial density of states (PDOS) of TM-$N_2O_2C_x$, (a)Sc-$N_2O_2C_x$, (b)Ti-$N_2O_2C_x$, (c)V-$N_2O_2C_x$, (d)Cr-$N_2O_2C_x$, (e)Mn-$N_2O_2C_x$, (f)Fe-$N_2O_2C_x$, (g)Co-$N_2O_2C_x$, (h)Ni-$N_2O_2C_x$, (i)Cu-$N_2O_2C_x$, (j)Zn-$N_2O_2C_x$. The dotted line denotes the Fermi level. The green, red and blue lines represent the 3d orbital of the metal atoms, the 2p orbital of the oxygen atoms, and 2p orbital of the Nitrogen atoms, respectively.

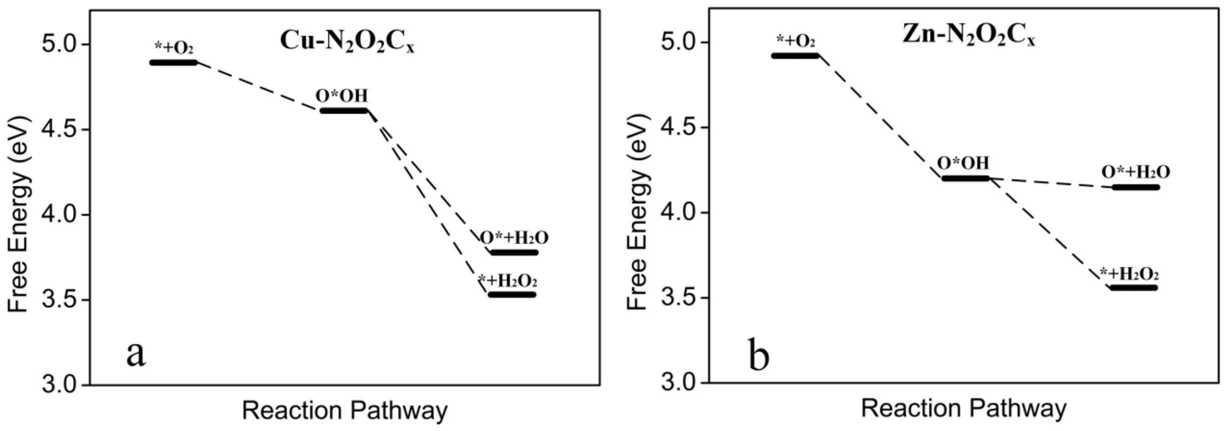

Figure S2. Free energy diagram of 2e- ORR on Cu-$N_2O_2C_x$ (a) and Zn-$N_2O_2C_x$ (b) under 0 V.

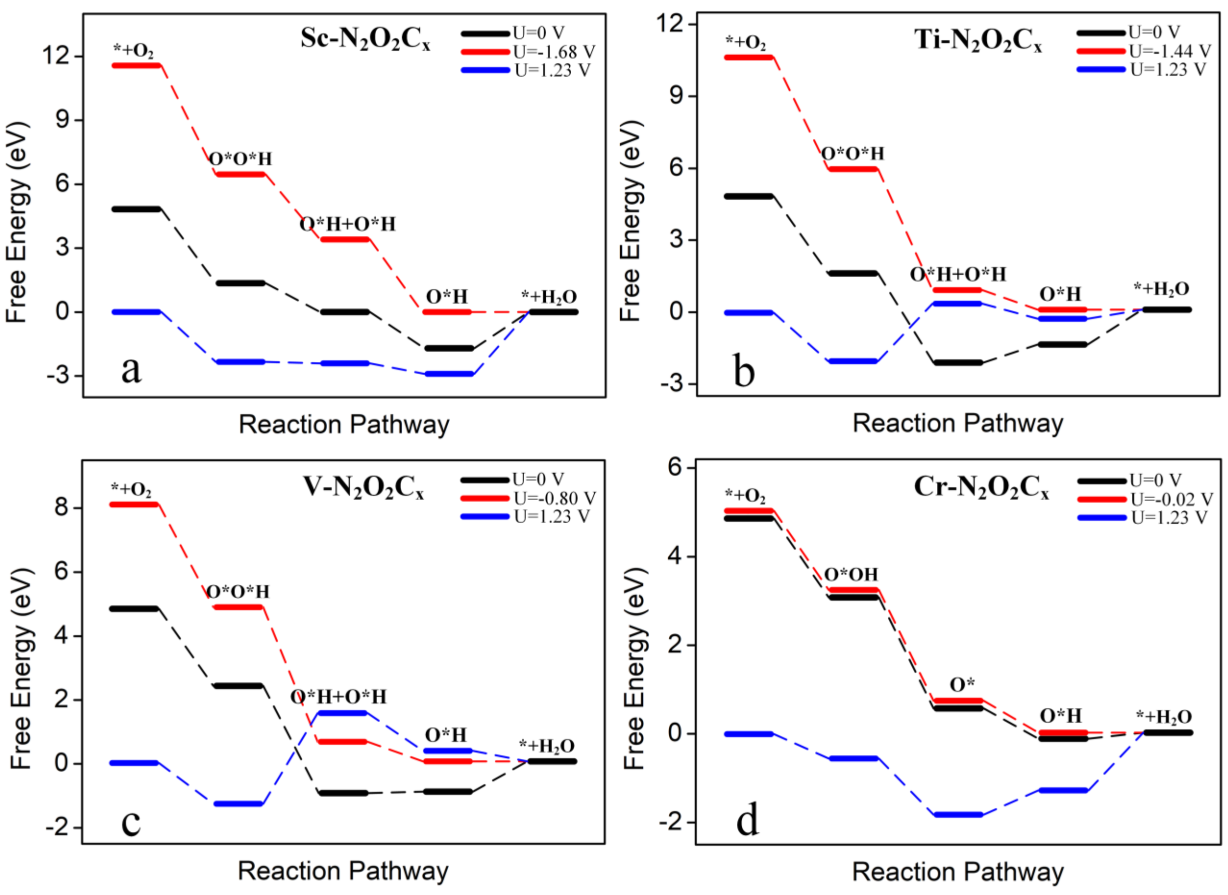

Figure S3. Free energy diagram of 4e- ORR on Sc-$N_2O_2C_x$ (a), Ti-$N_2O_2C_x$ (b), V-$N_2O_2C_x$ (c) and Cr-$N_2O_2C_x$ (d) under different potentials. The black line, blue line and red line represent U=0 V, U=limting potential and U=1.23 V, respectively.

Table S1 The lattice constants, top and side views of optimized crystal structures of TM-$N_2O_2C_x$; Hirshfeld charges on metal atoms (Q_{TM}), nitrogen atoms (Q_N) and oxygen atoms (Q_O) of TM-$N_2O_2C_x$ SACs, and Hirshfeld spin of the metal atoms, and the length of TM-N bond (R_{M-N}) and TM-O (R_{M-O}).

TM-$N_2O_2C_x$	Top-Side	Lattice constant	Q / e	Spin	R / Å
Sc-$N_2O_2C_x$	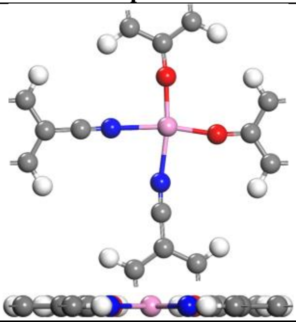	a=10.89 Å<br>b=10.88 Å<br>c=20.00 Å	Q_{TM}=0.684<br>Q_N=-0.191<br>Q_O=-0.259	0.000	R_{M-N}=2.213<br>R_{M-O}=1.962
Ti-$N_2O_2C_x$	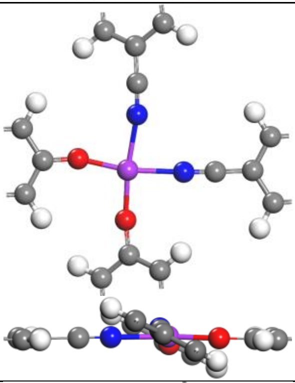	a=10.62 Å<br>b=10.61 Å<br>c=20.00 Å	Q_{TM}=0.371<br>Q_N=-0.166<br>Q_O=-0.226	1.446	R_{M-N}=2.120<br>R_{M-O}=1.893
V-$N_2O_2C_x$	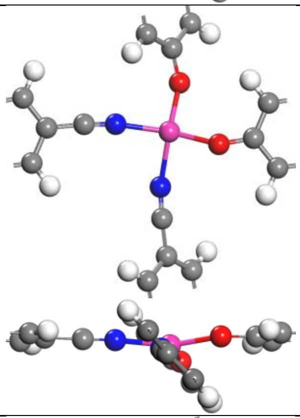	a=10.53 Å<br>b=10.53 Å<br>c=20.00 Å	Q_{TM}=0.341<br>Q_N=-0.150<br>Q_O=-0.220	2.536	R_{M-N}=2.025<br>R_{M-O}=1.933
Cr-$N_2O_2C_x$	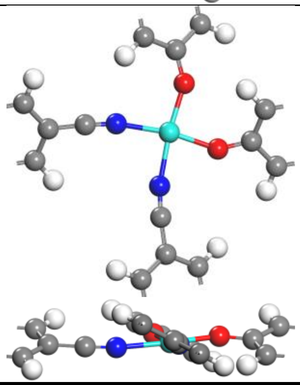	a=10.49 Å<br>b=10.50 Å<br>c=20.00 Å	Q_{TM}=0.495<br>Q_N=-0.173<br>Q_O=-0.262	3.569	R_{M-N}=2.029<br>R_{M-O}=1.941

$Mn\text{-}N_2O_2C_x$	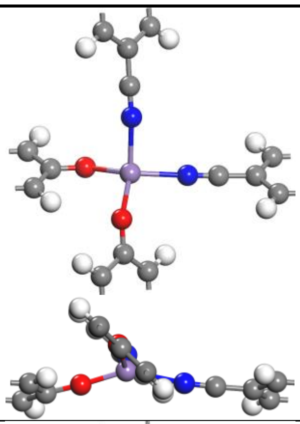	a=10.59 Å<br>b=10.59 Å<br>c=20.00 Å	Q_{TM}=0.437<br>Q_N=-0.154<br>Q_O=-0.257	4.504	$R_{M\text{-}N}$=2.182<br>$R_{M\text{-}O}$=2.010
$Fe\text{-}N_2O_2C_x$	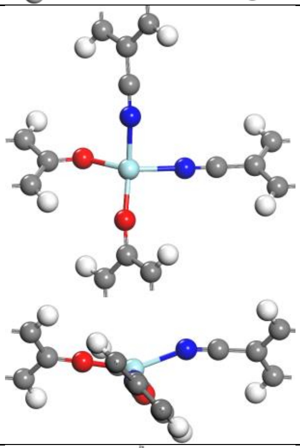	a=10.53 Å<br>b=10.56 Å<br>c=20.00 Å	Q_{TM}=0.345<br>Q_N=-0.149<br>Q_O=-0.241	3.478	$R_{M\text{-}N}$=2.079<br>$R_{M\text{-}O}$=1.907
$Co\text{-}N_2O_2C_x$	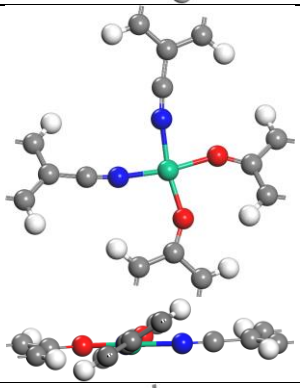	a=10.44 Å<br>b=10.42 Å<br>c=20.00 Å	Q_{TM}=0.165<br>Q_N=-0.109<br>Q_O=-0.201	0.816	$R_{M\text{-}N}$=1.876<br>$R_{M\text{-}O}$=1.888
$Ni\text{-}N_2O_2C_x$	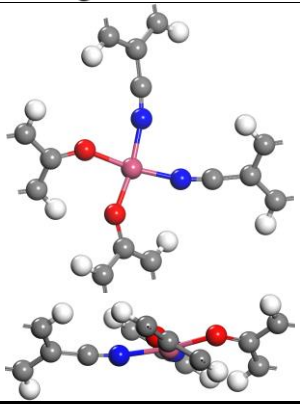	a=10.21 Å<br>b=10.15 Å<br>c=20.00 Å	Q_{TM}=0.153<br>Q_N=-0.106<br>Q_O=-0.190	0.000	$R_{M\text{-}N}$=1.858<br>$R_{M\text{-}O}$=1.863

Cu-$N_2O_2C_x$	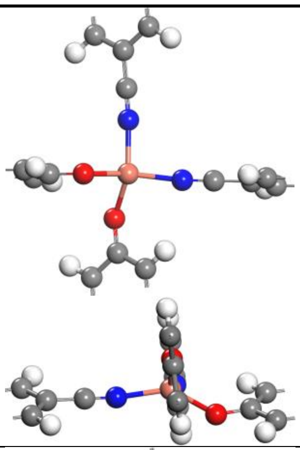	a=10.36 Å<br>b=10.34 Å<br>c=20.00 Å	Q_{TM}=0.410<br>Q_N=-0.169<br>Q_O=-0.255	0.416	$R_{M\text{-}N}$=2.005<br>$R_{M\text{-}O}$=1.981
Zn-$N_2O_2C_x$	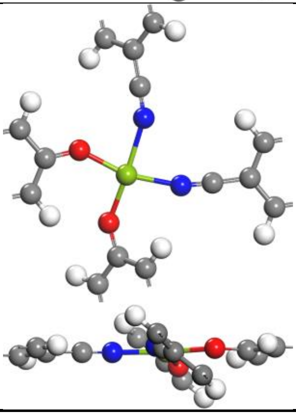	a=10.41 Å<br>b=10.45 Å<br>c=20.00 Å	Q_{TM}=0.553<br>Q_N=-0.183<br>Q_O=-0.264	0.000	$R_{M\text{-}N}$=2.055<br>$R_{M\text{-}O}$=1.974

Table S2 Computed formation energy (E_f) of TM-$N_2O_2C_x$, dissolution potential (U_{diss}) and number of transferred electrons (n) during the dissolution of metals. For comparison, the standard dissolution potential ($\mathbf{U°_{diss}}$) of metal atoms are also listed.

**TM-$N_2O_2C_x$**	**E_f / eV**	**$U°_{diss}$ / V**	**n / e**	**U_{diss} / V**
Sc-$N_2O_2C_x$	-6.46	-2.08	3	0.07
Ti-$N_2O_2C_x$	-4.40	-1.63	2	0.57
V-$N_2O_2C_x$	-3.33	-1.18	2	0.48
Cr-$N_2O_2C_x$	-3.24	-0.91	2	0.71
Mn-$N_2O_2C_x$	-2.72	-1.19	2	0.17
Fe-$N_2O_2C_x$	-2.18	-0.45	2	0.64
Co-$N_2O_2C_x$	-1.72	-0.28	2	0.58
Ni-$N_2O_2C_x$	-2.24	-0.26	2	0.86
Cu-$N_2O_2C_x$	-1.51	0.34	2	1.09
Zn-$N_2O_2C_x$	-3.62	-0.76	2	1.05

Table S3. The top and side views of the most stable adsorption state of O_2 on TM-$N_2O_2C_x$, as well as the corresponding adsorption energy ($E_{ads\text{-}O2}$).

TM-$N_2O_2C_x$-O_2	Top	Side	$E_{ads\text{-}O2}$ / eV
Sc-$N_2O_2C_x$-O_2	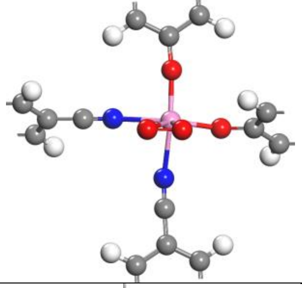	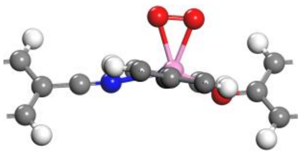	-2.85
Ti-$N_2O_2C_x$-O_2	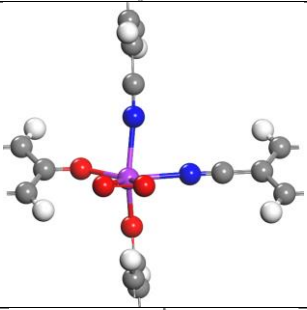	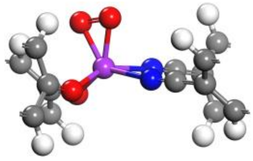	-4.22
V-$N_2O_2C_x$-O_2	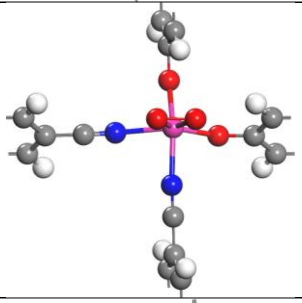	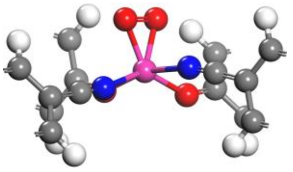	-3.03
Cr-$N_2O_2C_x$-O_2	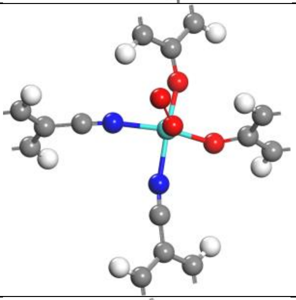	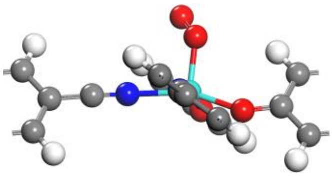	-1.33
Mn-$N_2O_2C_x$-O_2	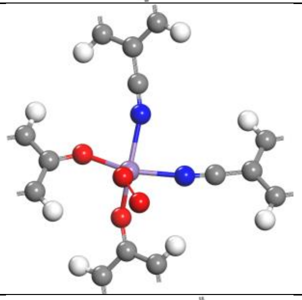	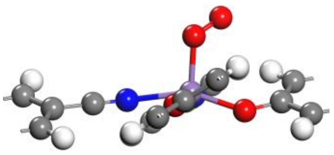	-0.87
Fe-$N_2O_2C_x$-O_2	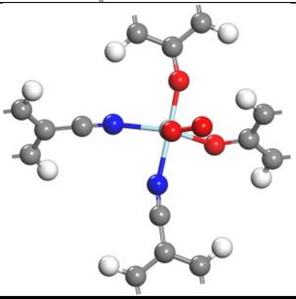	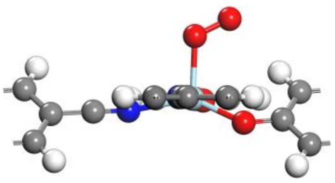	-0.77

Co-$N_2O_2C_x$-O_2	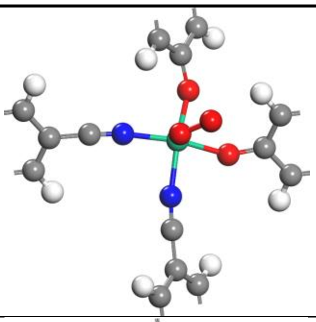	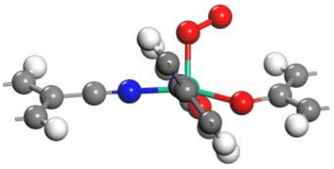	-0.83
Ni-$N_2O_2C_x$-O_2	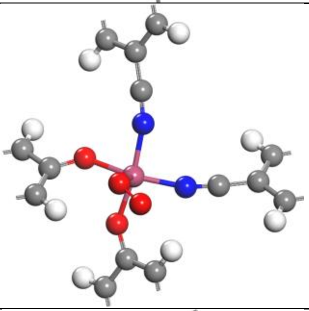	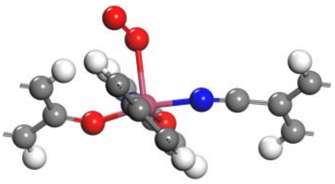	-0.42
Cu-$N_2O_2C_x$-O_2	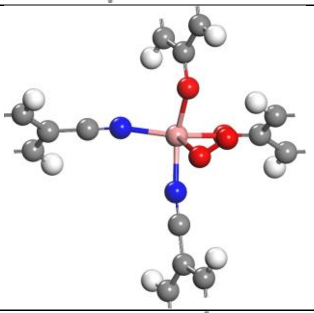	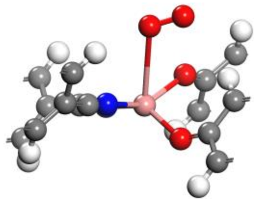	-0.39
Zn-$N_2O_2C_x$-O_2	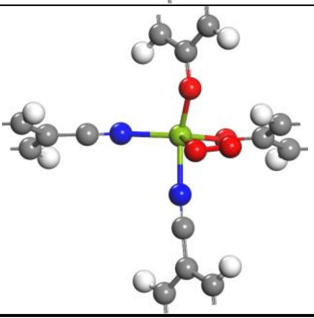	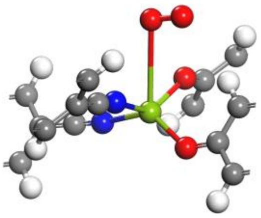	-0.57

Table S4. The calculated free energy corrections (Gc) for adsorbates: zero point energy (E_{ZPE}) and entropy correction (TS). The temperature of the reaction is 298.15k.

**Sc-$N_2O_2C_x$**	**E_{ZPE} (kcal/mol)**	**TS (eV)**	**G_c (kcal/mol)**
**O*O*H**	10.492	-0.089	12.567
**O***	2.206	-0.015	2.557
**O*H+O*H**	15.081	-0.104	17.471
**O*H**	9.334	-0.018	9.748

$Ti-N_2O_2C_x$	E_{ZPE} (kcal/mol)	TS (eV)	G_c (kcal/mol)
**O*O*H**	10.476	-0.082	12.367
**O***	2.289	-0.012	2.555
**O*H+O*H**	15.760	-0.080	17.616
**O*H**	9.407	-0.017	9.788

$V-N_2O_2C_x$	E_{ZPE} (kcal/mol)	TS (eV)	G_c (kcal/mol)
**O*O*H**	10.603	-0.082	12.498
**O***	2.266	-0.013	2.555
**O*H+O*H**	16.018	-0.074	17.717
**O*H**	9.301	-0.014	9.600

$Cr-N_2O_2C_x$	E_{ZPE} (kcal/mol)	TS (eV)	G_c (kcal/mol)
**O*OH**	10.205	-0.098	12.454
**O***	2.273	-0.013	2.567
**O*H**	9.403	-0.013	9.713

$Mn-N_2O_2C_x$	E_{ZPE} (kcal/mol)	TS (eV)	G_c (kcal/mol)
**O*OH**	10.452	-0.090	12.538
**O***	2.263	-0.013	2.559
**O*H**	9.403	-0.013	9.713

$Fe-N_2O_2C_x$	E_{ZPE} (kcal/mol)	TS (eV)	G_c (kcal/mol)
**O*OH**	9.920	-0.112	12.510
**O***	2.200	-0.012	2.481
**O*H**	9.322	-0.015	9.676

$Co-N_2O_2C_x$	E_{ZPE} (kcal/mol)	TS (eV)	G_c (kcal/mol)
**O*OH**	9.819	-0.125	12.454
**O***	2.244	-0.013	2.549
**O*H**	9.341	-0.014	9.670

$Ni-N_2O_2C_x$	E_{ZPE} (kcal/mol)	TS (eV)	G_c (kcal/mol)

**O*OH**	9.555	-0.094	11.733
**O***	2.323	-0.012	2.599
**O*H**	9.509	-0.013	9.804

**Cu-$N_2O_2C_x$**	**E_{ZPE} (kcal/mol)**	**TS (eV)**	**G_c (kcal/mol)**
**O*OH**	9.868	-0.121	12.649
**O***	2.323	-0.012	2.599
**O*H**	9.348	-0.014	9.676
**Zn-$N_2O_2C_x$**	**E_{ZPE} (kcal/mol)**	**TS (eV)**	**G_c (kcal/mol)**
**O*OH**	9.575	-0.099	11.867
**O***	2.198	-0.016	2.556
**O*H**	9.414	-0.015	9.757
